# Outcome after intensity modulated radiotherapy for anaplastic thyroid carcinoma

**DOI:** 10.1186/1471-2407-14-235

**Published:** 2014-04-02

**Authors:** Xiayun He, Duanshu Li, Chaosu Hu, Zhuoying Wang, Hongmei Ying, Yi Wu

**Affiliations:** 1 Department of Radiation Oncology, Department of Oncology, Fudan University Shanghai Cancer Center, Shanghai Medical College, Fudan University, Shanghai 200032, China; 2 Department of Head and Neck Surgery, Department of Oncology, Fudan University Shanghai Cancer Center, Shanghai Medical College, Fudan University, Shanghai 200032, China

**Keywords:** Anaplastic thyroid carcinoma, ATC, Intensity modulated radiotherapy, Locoregional control, Distant metastases

## Abstract

**Background:**

Anaplastic thyroid carcinoma (ATC) is a malignancy with one of the highest fatality rates. We reviewed our recent clinical experience with intensity modulated radiotherapy (IMRT) combined with surgery and chemotherapy for the management of ATC.

**Methods:**

13 patients with ATC who were treated by IMRT in our institution between October 2008 and February 2011, have been analyzed. The target volume for IMRT was planned to include Gross tumor volume (GTV): primary tumor plus any N + disease (66 Gy/33 F/6.6 W), with elective irradiation of thyroid bed, bilateral level II through VI and mediastinal lymph nodes to the level of the carina (54-60 Gy). Seven patients received surgical intervention and eleven patients had chemotherapy.

**Results:**

The median radiotherapy dose to GTV was 60 Gy/30 fractions/6 weeks. The median survival time of the 13 patients was 9 months. The direct causes of death were distant metastases (75%) and progression of the locoregional disease (25%). Ten patients were spared dyspnea and tracheostomy because their primary neck lesion did not progress.

**Conclusion:**

The results showed that IMRT combined by surgery and chemotherapy for ATC might be beneficial to improve locoregional control. Further new therapies are needed to control metastases.

## Background

Anaplastic thyroid carcinoma (ATC) is a rare malignant tumor and accounts for <5% of all thyroid cancers in incidence, but is responsible for up to 50% of all thyroid cancer deaths. ATC is very aggressive and can invade directly into adjacent organs, such as the trachea, larynx, esophagus, blood vessels and muscle, resulting in suffocation, vocal cord paralysis, dyspnea and/or dysphagia. Most patients die a few months after diagnosis because of uncontrolled neck mass and metastases, and long-term survivors are rare [1-4]. In the dead patients, dyspnea was the most common cause due to extensive neck mass, and another cause was metastases [5]. A tracheotomy may sometimes be performed to solve airway obstruction, but which also deteriorated the quality of life in the patients.

Because the prognosis for ATC is so poor, all ATC are classified as stage IV according to International Union Against Cancer (UICC) staging, regardless of tumor size, nodal status, and either absence or presence of distant metastasis. Combined modality therapy with surgery, chemotherapy and two dimensional or conformal radiotherapy, have been adopted for ATC [6-9], but the results have not been satisfactory. Lim et al. [8] reviewed the treatment outcome of 13 consecutive patients retrospectively, of which five, two, and six patients had stage IVA, IVB, and IVC disease respectively. They received 3-dimensional conformal radiotherapy and chemotherapy, plus surgery, the median progression-free survival and overall survival were 2.8 and 3.8 months respectively. Considering that patients with ATC showed poor prognosis despite multimodality treatment, an effective mode of treatment warranted further study. Intensity modulated radiotherapy (IMRT) is an advanced form of three dimensional conformal radiotherapy, conforming high dose to tumor with low dose to normal tissues, so that a good therapeutic ratio can be achieved. The paper report the outcomes for the first 13 consecutive patients treated with IMRT combined with surgery and chemotherapy.

## Methods

The study was approved by our institution’s ethics committee (Fudan University Shanghai Cancer Center Institutional Review Board, reference number 090371-5). We analyzed the outcomes of 13 ATC patients treated with IMRT in our institution between October 2008 and February 2011. There were 10 males and 3 females, whose median age was 62 (range, 43-75 years). The diagnosis of six patients with ATC was based on aspiration biopsy with addition of immunohistochemistry and the other seven were diagnosed histologically by biopsy or surgery. Five patients had evidence of distant metastases at presentation. The sites of distant metastases were lung (3 patients), bone (1 patient), and at multiple sites (1 patient). The clinical characteristics of the thirteen patients are listed in Table 1. Initial evaluation included physical examination, routine labs, computed tomography (CT) scans of the chest and neck, abdominal ultra-sonography, and bone scintigram. Additional investigations were performed only on those with suspicious findings. ATC patients can be divided into 3 subgroups: (1) stage IVA, a tumor limited to the thyroid gland; (2) stage IVB, a tumor beyond the thyroid capsule; and (3) stage IVC, tumors exhibiting distant metastasis. According to this staging system, of our 13 patiens, 8 were IVB, and 5 wereIVC (Table 1).

**Table 1:** Patients characteristics

***Number of patients (%)***	***13***
**Median age at diagnosis** (years)	62(43-75)
**Gender**	
Female	3(23.1)
Male	10(76.9)
Female/male ratio	0.3
**KPS**	
70	9(69.2)
80	4(30.8)
**Diagosis**	
Histopathology	7(53.8)
FNA	6(46.2)
**Stage**	
IVB	8(61.5)
IVC	5(38.5)

KPS: Karnofsky performance status; FNA: fine needle aspiration.

In our institution we have tried IMRT combined with chemotherapy and/or surgery for these 13 ATC patients. All patients were treated in the supine position using thermoplastic mask and received IMRT with 6 MV X ray. The simulation CT scans were done using 5 mm slices from the head to the level of diaphragm. Gross tumor volume (GTV) included the primary tumor and metastatic lymph nodes found in clinical and imaging examinations. Clinical target volume (CTV) included thyroid area and regional lymph nodes, with elective treatment of bilateral level II through VI and mediastinal lymph nodes to the level of the carina. High-risk clinical target volume was defined as thyroid bed plus regional lymph node involved by tumor. All patients were irradiated 1 fraction daily, 5 days per week. A dose of 66 Gy/33 fractions/6.6 weeks was prescribed to the planning target volume (PTVg), defined as the GTV plus 0.5 cm margin. A dose of 60 Gy was prescribed to the high-risk clinical target volume plus 0.5 cm margin. A dose of 54 Gy was prescribed to the low-risk clinical target volume.

After treatment, patients were follow-up every 3 months for the first 2 years, then every 6 months for the following years. All patients were followed until death or time of analysis. The survival time was determined from the date of treatment to the time of death or the date of the most recent follow-up examination.

## Results

### Radiotherapy

Table 2 summarizes the treatment and outcomes of the 13 patients. The median total dose of GTV was 60 Gy (range, 40-66 Gy). The median overall radiation therapy treatment time was 42 days (range, 28-49 days), and the median number of fractions was 30 (range, 20-33 fractions). Nine patients (75%) completed radiotherapy at a dose ≥ 60 Gy. Two patients gave up radiotherapy due to dysphagia at dose of 40 Gy and 54 Gy. One month later, the symptom disappeared, computed tomography (CT) scan of the two patients demonstrated shrinkage of tumor. One patient at stage IVC developed enlargement of lung and head lesion during concurrent radiochemotherapy, although the neck lesion shrunk after 50 Gy. The patient stopped radiotherapy, and received multi-agent chemotherapy (GP) instead. One patient appeared to have headache during IMRT, MRI scan showed brain metastasis and radiotherapy of thyroid area and regional lymph nodes was stopped at 58 Gy.

**Table 2:** treatment and result

***Type of surgery (%)***	
*R0*	*1(7.7)*
*R2*	*1(7.7)*
*Biopsy*	*3(23.1)*
*Biopsy + tracheotomy*	*2(15.4)*
**Type of Chemotherapy** (%)	
Neo	1(7.7)
Con	3(23.1)
Adj	1(7.7)
Neo + Adj	1(7.7)
Neo + Con	2(15.4)
Con + Adj	2(15.4)
Con + Neo + Adj	1(7.7)
**RT** (Gy)	
≤50	2(15.4)
51-59	2(15.4)
≥60	9(69.2)
**Median follow-up** (months)	9(2-36)
**Treatment response** (%)	
CR	2(15.4)
PR	1(7.7)
SD	7(53.8)
PD	3(23.1)
**DM** (%)	
Yes	7(53.8)
None	6(46.2)
**State** (%)	
Alive	5(38.5)
Dead	8(61.5)

R0, no residual tumor; R2, macroscopic residual tumor; Neo, neoadjuvant; Con, concurrent; Adi, adjuvant; CR, complete response; PR, partial response; SD, stable disease; PD, progressive disease; DM, distant metastasis.

### Chemotherapy

Eleven patients received chemotherapy, eight of whom were given concurrent chemotherapy with cisplatin (DDP) 35 mg/m^2^/week in six patients, paclitaxel 70 mg/m^2^/week in one patient, and paclitaxel 135 mg/m^2^ on d1 and cisplatin 25 mg/m^2^ on d1-3, repeated on day 28 in one patient. Eight patients were given neoadjuvant or adjuvant chemotherapy every 3 weeks for 2 cycles. Of these, four were given gemcitabine and cisplatin, two were given docetaxel and cisplatin, one had paclitaxel and cisplatin, and fluorouracil and cisplatin in one. The doses for the drugs are as follows: cisplatin 25 mg/m^2^ on d1-3, and gemcitabine 1000 mg/m^2^ on days 1 and 8, fluorouracil 500 mg/m^2^, paclitaxel 135 mg/m^2^, docetaxel 60 mg/m^2^, every 3 weeks. Adjuvant chemotherapy with the same regimen was administered after 28 days after the end of radiotherapy.

### Surgery

Surgical intervention included complete resection of cervical nodes in one patient with lung metastasis, partial resection in one patient, biopsy only in five, and tracheotomy in two.

### Toxicities

There were no treatment-related deaths. During IMRT, mucositis of pharynx and esophagitis were the most common acute treatment toxicities. All patients developed mucositis/esophagitis: RTOG grade I in eleven patients and grade II in two. Six patients needed nutritional support intravenously for mucosal reaction. The median duration was 6 days (3 - 9 days), but none of them required tube feeding support. Radiotherapy was given up in two patients due to esophagitis (grade II). One month later, the esophagitis disappeared and the tumor was shown to have shrunk as seen by CT scan. Main side effects attributed to chemotherapy were hematological and digestive toxicity. Grade 3 and 4 neutropenia were observed in three patients, and grade 3 thrombocypenia was observed in one. All patients had grade I/II nausea and vomiting. There was no late adverse effect of therapy (≥ grade II), such as pneumonitis, esophageal stenosis, fibrosis in neck.

### Local control, distant metastasis, and survival

At a median follow-up duration of twelve months (range 2 to 36 months), mean survival was 11 months and median survival was 9 months. At the latest follow-up, five patients were alive and have been followed for 8, 8, 12, 15, 36 months, respectively. The locoregional disease were in complete remission in 2 patients, partial remission in 1 patient, was stabilized in 7 patients and there was progressive disease in 3 patients. Distant metastases were found in seven patients, and the sites were lung (1 patient), bone (1 patient), brain (1 patient) and multiple sites (4 patients). Eight patients died 2, 4, 7, 8, 9, 9, 12, and 16 months after treatment respectively. The direct causes of death were distant metastases (6 patients) and locoregional failure (2 patients).

## Discussion

Anaplastic thyroid carcinoma has a low incidence but is highly lethal, so there have been no large phase III trials. The most available evidence is derived from retrospective series, which suggest that a combination of surgery, radiotherapy, and chemotherapy may be useful. One hundred patients with ATC at Ito Hospital in Tokyo between 1993 and 2009 were reviewed, most of whom were in stage IVC (58%) and IVB (31%). The 1-year survival rates of IVB, and IVC were 24.8%, 8.2% respectively [10]. Derbel O. et al treated 44 patients with ATC between 1996 and 2010, complete response, partial response, and progression after treatment were 31.8%, 18.2%, and 50.0%. The median survival of the 44 patients was 8 months [11].

It is known that the total radiation dose is related to local control, and the failure of locoreginal control of ATC leads to dyspnea, which would need tracheostomy. Swaak-Kragten AT et al. reported a retrospective study on 75 patients with ATC, the median overall survival for patients who had received a total dose of > 40 Gy versus ≤ 40 Gy, was 5.4 and 1.7 months, respectively (P < 0.001) [12]. Another study indicated that median survival of patients receiving 40 Gy or more (n = 24) was longer than those with less than 40 Gy (n = 34) (9 vs 3 months) [13]. Compared with 2D, IMRT provides a more conformal high dose with improved homogeneity to the gross disease and high-risk areas, while lowering the dose to normal organs at risk, including spinal cord, epiglottis false/true vocal cords, pharyngeal constrictors and esophagus. Our results compared to other report supported the hypothesis that IMRT may improve ATC outcome, especially local control. Foote RL et al reported that ten patients (40%) had regionally confined ATC (stag IVA in 7 patients, IVB in 3 patients) were treated by IMRT combined with individualized surgery (where feasible), and chemotherapy. The target volume included any residual cancer within the thyroid bed and/ regional lymph nodes, with elective treatment of bilateral level II- VI, mediastinal lymph nodes to the level of the carina, and received 57.6-70 Gy. Five patients (50%) are alive and disease-free, and overall survival at 1 and 2 years was 70% and 60%, respectively [14]. Our preliminary results of IMRT showed: the tolerance of patients was significantly improved, almost all patients received higher dose of radiation (> 54 Gy). Only two patients received 40 Gy and 54 Gy respectively and they gave up the radiotherapy because of esophagitis, but after one month their dysphagia improved. Another patient with lung metastasis received 50 Gy and stopped concurrent radiochemotherapy (cisplatin). Instead he was given combined chemotherapy, the bigger neck mass became smaller and dyspnea was relieved. Higher dose brought neck mass under control, 76.9% (10/13) patients obtained locoregional control (including complete and partial response and stable disease), thus avoiding tracheostomy due to breathing difficulties, which significantly improved the patient’s quality of life and survival. The present study of IMRT in ATC showed an apparent improvement in median survival of 4-8 months, although the number of patients in this series is modest.

For patients with ATC, the main causes of death were failure of local control and distant metastases. Multivariate analysis demonstrated that age ≥ 70 years, white blood cell (WBC) ≥ 10,000 mm^3^, extra-thyroidal invasion, distant metastases, avoidance of multimodality therapy, and radiation therapy with a dose of < 40 Gy were risk factors for poorer survival [6,15], but the most direct cause of death is upper airway obstruction as reported by Tashima L et al. [5]. In his study of 33 patients, dyspnea was the only independent factor affecting the survival. For ATC, the opportunity of complete surgery is rare because of its extensive invasion and distant metastases. Tracheotomy procedures have to be performed to resolve dyspnea caused by compression by the neoplastic mass or bilateral laryngeal nerve palsy, but it was not shown to significantly increase survival time and may sacrifice quality of life [5]. In this series of patients treated with IMRT, direct causes of death were distant metastases (6/8 patients) and locoregional progression (2/8 patients). Most of the patients present at diagnosis with metastatic disease, but almost all patients developed new metastasis during the rapid course of the disease. Chemotherapy has been studied for many years, but it is still unclear as to which systemic therapy is the best. Over the past few years, the most used agent in ATC is doxorubicin, cisplatin, 5-fluorouracil, and mitoxantrone, but the results of these drugs were disappointing. In recent years, new drugs including paclitaxel, gemcitabine have been used to try to increase overall survival in patients with ATC, but until now they do not seem to improve the outcomes significantly [16-20]. In ATC patients reported at stage IVB (n = 9), IVC (n = 4), induction chemotherapy by weekly paclitaxel is a promising therapeutic strategy and responders can be expected to achieve long-term survival, compared to that of ATC patients at stage IVB treated without paclitaxel (n = 50). However, no significant difference of overall survival was observed in patients at stage IVC patients with or without weekly induction paclitaxel (n = 13) [18]. In our series, cisplatin was combined with 5-fluorouracil, paclitaxel, and gemcitabine respectively, but there was no clear correlation between the specific chemotherapy administered and its outcome.

The main toxicity of radiotherapy was dysphagia and esophagitis, which may have made patients quit the radiotheraphy. Five patients with ATC have been treated at the Tom Baker Cancer Centre, radiation therapy (RT) was delivered in two phase using a three-dimensional (3D) conformal technique, 60% of patients needed tube feeding and 20% required tracheostomy because of dysphagia or dyspnea [21]. Troch M et al. reported that a total of six patients with ATC received standard external beam RT of 60 Gy in 30 fractions alone with docetaxel, all patients developed severe esophagitis, which resulted in inability to swallow food and and thereafter needed parenteral nutrition. Only three patients received the planned 60 Gy, other three patients discontinued at 40, 44, and 50 Gy because of side effects [22]. As opposed to these results, the side effect in our study of IMRT was mild: none of the patients required tube feeding support or tracheostomy during radiotherapy. There are limitations with our analyses: the dataset is relatively small considering the number of variables that were evaluated, thus limiting the impact of our analyses.

## Conclusions

In summary, our consecutive series of stage IVB, and IVC ATC patients treated with IMRT combined multimodality treatment demonstrates encouraging long-term survival with acceptable toxicity. The main direct cause of death for ATC patients was distant metastases instead of airway obstruction. New therapies need further investigation.

## Competing interests

The authors declare that they have no competing interests.

## Authors’ contributions

XH: Study design, data collection, data analysis, manuscript preparation, manuscript review. DL: Study design, data collection, data analysis, manuscript preparation, manuscript review. CH: Study design, manuscript preparation and review. ZW: Study design and data collection. HY: Study design and statistical analysis. YW: Reviewed the draft, provided comments or revisions, and approved the final manuscript. All authors read and approved the final manuscript.

## Pre-publication history

The pre-publication history for this paper can be accessed here:

http://www.biomedcentral.com/1471-2407/14/235/prepub
